# Prognostic value of lncRNA SOX2OT for Chinese cancer patients: A meta-analysis

**DOI:** 10.1371/journal.pone.0176889

**Published:** 2017-05-10

**Authors:** Xuran Jing, Jieru Lin, Hongwei Wang, Liyuan Tian, Runhua Tian, Yunyuan Zhang, Xian Chen, Jinyu Zhang

**Affiliations:** 1 Medical college of Qingdao university, Qingdao, Shandong, China; 2 Department of Respiratory and Critical Care Medicine, Guizhou Provincial People's Hospital, Guiyang, Guizhou, China; 3 Department of Clinical Laboratory, the Affiliated Hospital of Qingdao University, Qingdao, Shandong, China; 4 Department of Biochemistry and Molecular Biology, College of Medical, Qingdao University, Qingdao, Shandong, China; University of South Alabama Mitchell Cancer Institute, UNITED STATES

## Abstract

SOX2OT has been demonstrated to be aberrantly expressed in several types of cancer and maybe serve as a prognostic marker for cancer patients. However, most individual studies have been limited by small sample sizes and controversial results. Therefore, the present meta-analysis was conducted to analyze available data to reveal the potential clinical application of SOX2OT on cancer prognosis, tumor progression, distance metastasis and lymph node metastasis. Up to February 20, 2017, literature collections were conducted by comprehensive searching electronic databases, including Cochrane Library, PubMed, Embase, BioMed Central, Springer, ScienceDirect, ISI Web of Knowledge, together with three Chinese databases: China National Knowledge Internet (CNKI), Weipu and Wanfang. The hazard ratios (HR) with 95% confidence interval (95% CI) were calculated to assess the strength of the association. Five studies with a total of 481 cancer patients were included in the present meta-analysis. The results indicated that elevated SOX2OT significantly predicted unfavorable overall survival (OS) (HR = 2.44, 95% CI: 1.75–3.39, *P*<0.0001) and tumor progression (III/IV vs. I/II: HR 1.62, 95%CI: 1.30–2.02, *P*<0.0001), but failed to predict distant metastasis (HR: 3.30, 95%CI: 0.74–14.61, *P* = 0.12) and lymph node metastasis (HR: 1.29, 95% CI: 0.87–1.91, P = 0.21). The results revealed that SOX2OT expression level was an independent prognostic biomarker for OS and tumor progression in Chinese cancer patients.

## Introduction

Due to the increasing mobility and mortality, cancer is still a major problem for public health all over the world[1]. In 2017, 1,688,780 new cancer cases and 600,920 cancer deaths are projected to occur in the United States[2]. The long term survival rate remains low in various types of cancer, and numerous scientists are dedicated to searching new potential biomarkers for early diagnosis and accurate prognosis for cancer patients[3, 4].

Long noncoding RNAs (lncRNAs), which constitute the majority of transcripts encoded by the human genome, are non-protein coding RNA molecules greater than 200 nucleotides in length[5]. With the rapid development of genome-wide analysis technology, exponential growth of studies have been presented to suggest that lncRNAs are important regulatory molecules at every level of cellular physiology, including alternative splicing, cell cycle control, chromatin modification, dosage compensation, gene imprinting, genome rearrangement, and nuclear-cytoplasmic trafficking[6–8]. Moreover, lncRNAs have attracted considerable attention as vital modulators in carcinomas due to the potential role of lncRNAs in tumor development, progression, and metastasis[9–11].

SOX2 overlapping transcript (SOX2OT), generating at least six transcript variants, is highly expressed in embryonic stem cells. Recently, increasing clinical studies indicated that elevated expression of SOX2OT was closely linked with poor prognosis and high risk of cancer metastasis in many types of carcinomas. However, most individual studies assessing the implication of SOX2OT expression levels in cancer have been limited by small sample sizes and controversial results. To our knowledge, no systematic meta analysis has been conducted to evaluate the relationship between SOX2OT expression and the relevant clinical outcomes. Accordingly, it is necessary to perform a meta analysis to elucidate the clinical feasibility of SOX2OT as a putative biomarker candidate by systematically summarizing all eligible articles.

## Material and methods

### Search strategy and literature selection

Articles up to February 20, 2017, which related to the lncRNA SOX2OT as a potential eligible biomarker for the prognosis of cancer patients, were comprehensive searched in several electronic databases, including Cochrane Library, PubMed, Embase, BioMed Central, Springer, ScienceDirect, ISI Web of Knowledge, together with three Chinese databases: China National Knowledge Internet (CNKI), Weipu and Wanfang. Publications with the following keywords were included: (‘‘long noncoding RNA- “OR ‘‘lnc RNA-” OR ‘‘noncoding RNA-” OR ‘‘SOX2OT” OR ‘‘SOX2-OT”) AND (‘‘cancer” OR ‘‘carcinoma” OR ‘‘tumor” OR ‘‘neoplasm”) AND (“prognosis” or “prognostic” or “survival” or “metastasis”). The reference lists of primary publications were manually viewed to obtain additional relevant articles.

### Inclusion and exclusion criteria

Inclusion criteria: 1) Definite diagnosis or histopathological confirmed for patients with cancer; 2) Articles investigating the expression pattern of SOX2OT in any malignant tumor; 3) Sufficient information for the computation of hazard ratios (HR) and corresponding 95% confidence intervals (CI).

Exclusion criteria: 1) Basic research; 2) Studies of non dichotomous SOX2OT expression or absence of survival outcome; 3) Multiple duplicate articles about a study, excluding earlier and smaller sample data; 4) Animal experiments, case reports, correspondences, editorials, expert opinions, letters, review articles and talks without original data.

### Data extraction and quality assessment

Two authors (XRJ and JRL) reviewed each eligible article and extracted the data independently. All of the differences and contradictions were resolved by a third investigator. The major information from each enrolled study was extracted: (1) last name of first author, publication year, study design, country, cancer type, total cases, stage, follow-up time; (2) SOX2OT assessment method and specimen resources; (3) hazard ratio (HR) with 95% confidence interval (CI) of SOX2OT for overall survival, patient number for TNM stage and progression, lymph node metastasis or distant metastasis. If univariate and multivariate analysis were both provided by the eligible studies, multivariate analysis was preferred because multivariate values have higher precision on interpreting confounding factors.

### Quality assessment

MOOSE and PRISMA checklist were used to evaluate the quality of included publications [12] (S1 and S2 Tables).The Newcastle-Ottawa scale (NOS) for cohort studies was also conducted to assess the quality of involved studies (S3 Table). Two authors independently performed the quality assessments (XRJ and JRL). The NOS contains three categories (selection, comparability, and outcome) and eight items. In the selection and outcome categories, a quality research item received one star, and a comparable category could receive at most two stars. The quality assessment values ranged from 0 to 9 stars.

### Statistical analysis

The impact of SOX2OT expression on overall survival, TNM stage and progression, distance metastasis and lymph node metastasis was examined by HRs and 95% CIs. An observed HR >1 indicated poorer prognosis in patients with elevated SOX2OT expression and should be statistically significant when the 95% CI did not overlap with 1. The random-effects model was conducted to analyze the relationship between SOX2OT expression and clinical outcomes when calculated *I*^2^>50%[13–15]. Probable publication bias was examined by a funnel plot and Begg’s bias test[16]. P values <0.05 was considered statistically significant. All statistical analyses were performed using Stata SE 12.0 (Stata Corporation) and RevMan 5.3 software.

## Results

### Included literatures

**Fig 1:**
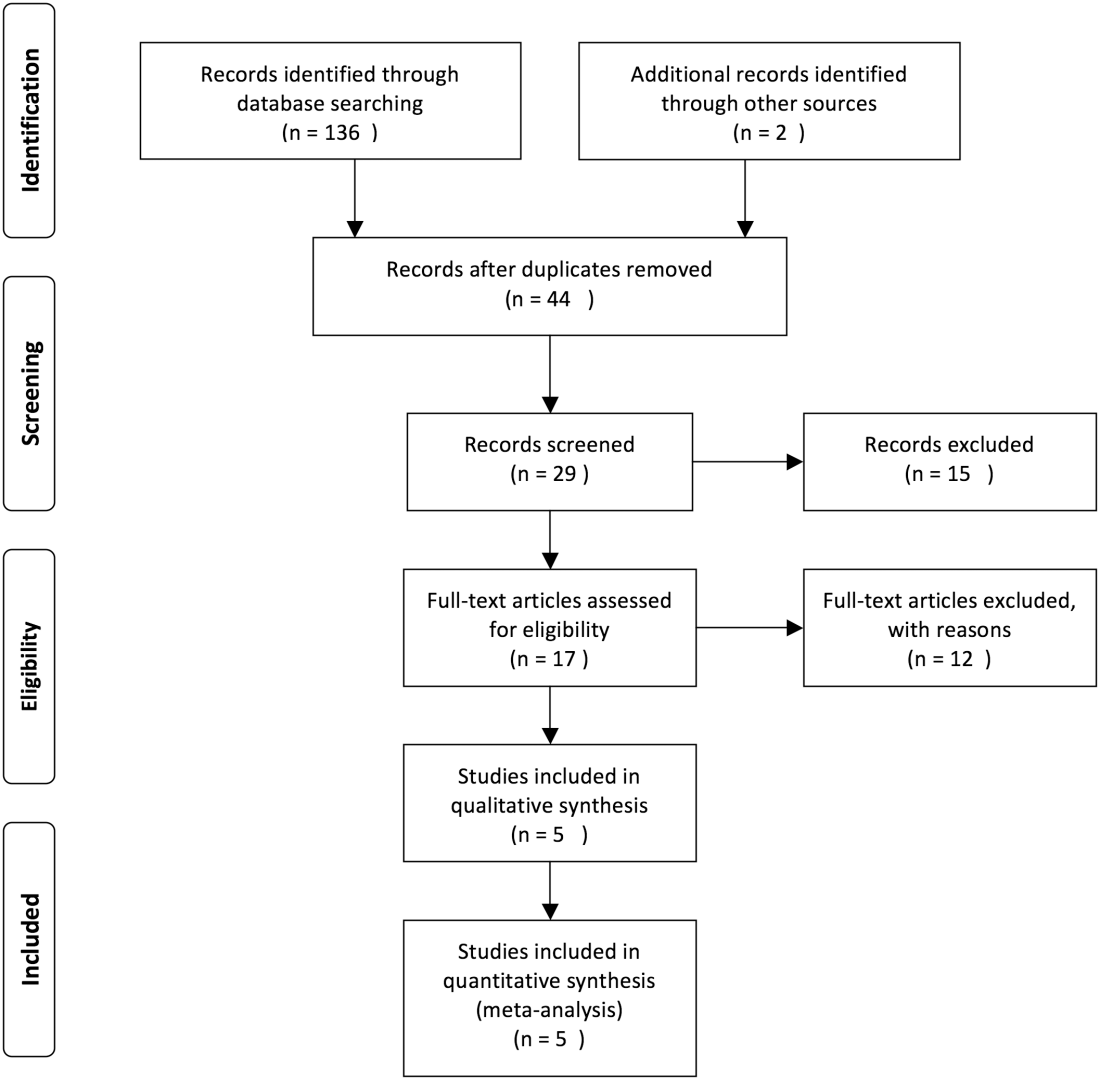
Flow diagram of the study search and selection process.

Fig 1 presented the literature screening and study selection processes. The initial search from electronic databases retrieved a total of 138 studies concerning the prognosis or metastasis of SOX2OT and cancer. After carefully screening the titles and abstracts, 121 articles were excluded because they were basic studies, letters, duplicate articles, reviews, or irrelevant to the present study. Full texts of the remaining 17 articles were further reviewed and assessed, and 12 articles were then removed because SOX2OT was not a dichotomic variable in the original studies. Ultimately, 5 articles were included in the current analysis[3, 17–20].

### Characteristics of the enrolled studies

The main characteristics of eligible articles were summarized in Table 1. In summary, the sample sizes of these articles ranging from 38 to 132. All of the 421 patients were divided into high or low SOX2OT group according to the SOX2OT measurement results. Four of five studies were from China and related to 4 kinds of carcinomas, including lung cancer, hepatocellular cancer, gastric cancer and breast cancer. Notably, the cut-off values were different, with median was applied in most articles.

**Table 1:** Summary of the five included studies.

Study	Origin of population	Study design	Disease	N	Stage	TUG1 assay	Survival analysis	Metastasis analysis	Hazard ratios	Follow-up Months
Hou 2014	China	R	LC	47	I/II, III/IV	qRT-PCR	OS	LNM	HR/K-M	60
Shi 2015	China	R	HC	84	I/II, III/IV	qRT-PCR	OS	NA	HR/K-M	60
Zhang 2016	China	R	GC	132	I/II, III/IV	qRT-PCR	OS	LNM/DM	HR/K-M	96
Zou 2016	China	R	GC	120	I/II, III/IV	qRT-PCR	OS/DFS	LNM/DM	HR/K-M	60
Iranpour 2016	Iran	R	BC	38	I/II, III/IV	qRT-PCR	NA	LNM	NA	NA

Study design is described as retrospective (R); LC, lung cancer; HC, hepatocellular carcinoma; GC, gastric cancer; BC, breast cancer; DM, distant Metastasis; LNM, Lymph Node Metastasis.

### Meta analysis results

**Fig 2:**
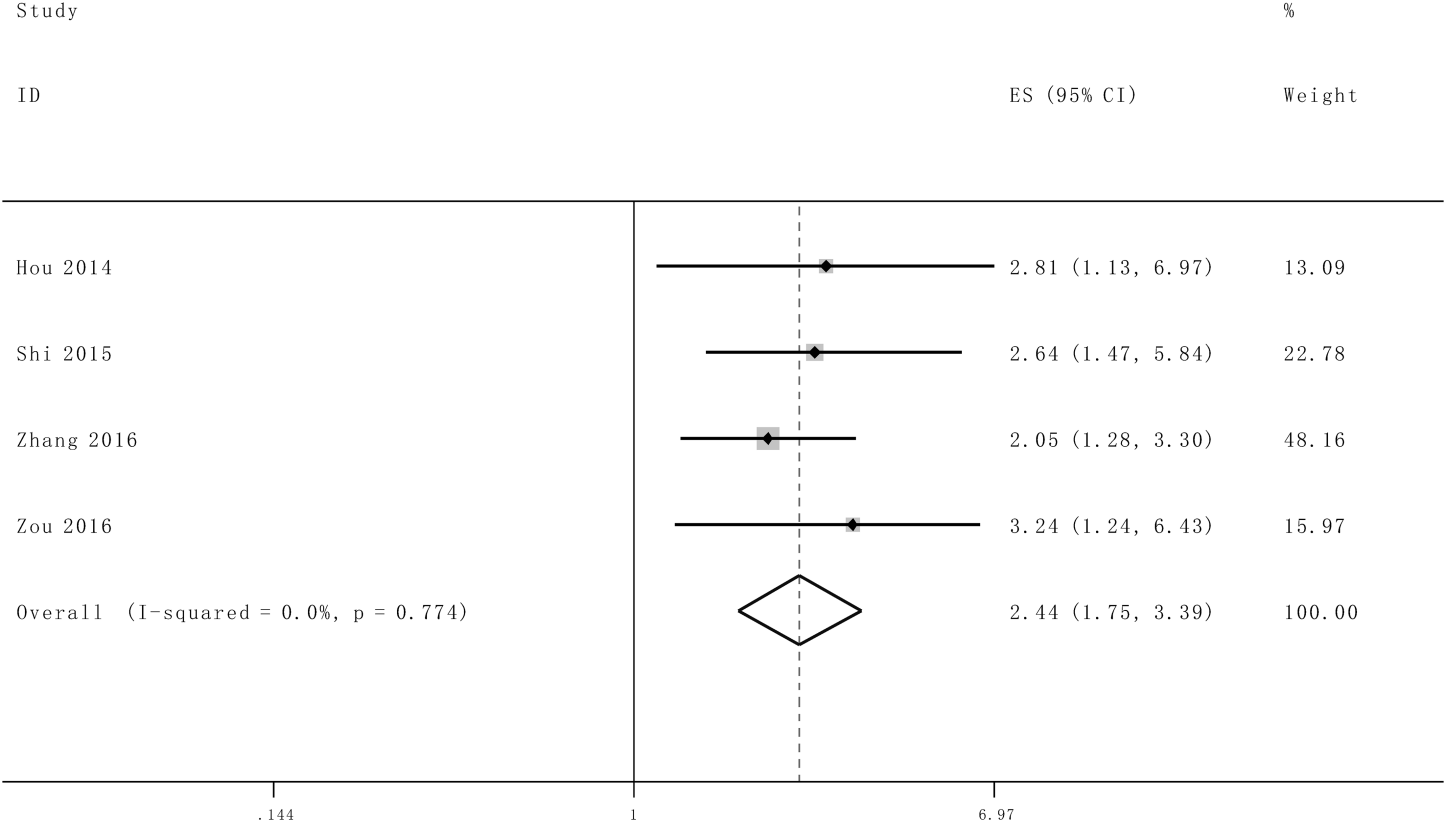
Forest plot for the association between SOX2OT expression with overall survival (OS).

**Fig 3:**
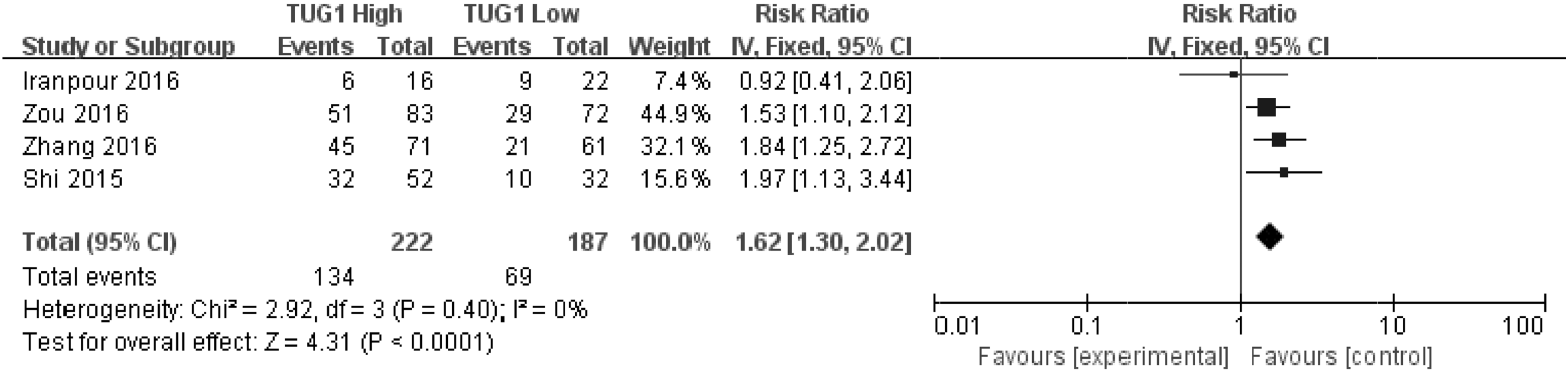
Forest plot for the association between SOX2OT expression with TNM stage (III/IV vs. I/II (A) and II/III/IV vs. 0/I (B)).

As indicated in Fig 2, *I*^2^ values for OS was 0.0%. Therefore, a fix effects model was employed to analysis the pooled HR and its 95% CI because there is no existence of significant heterogeneity among those 5 studies which involved in OS and tumor progression analysis. Enforced SOX2OT expression was predictive of unfavorable OS (HR = 2.44, 95% CI: 1.75–3.39, *P*<0.0001) and tumor progression (III/IV vs. I/II: HR = 1.62, 95% CI: 1.30–2.02, *P*<0.0001) in various carcinomas with multivariate analysis, respectively (Figs 2 and 3).

**Fig 4:**
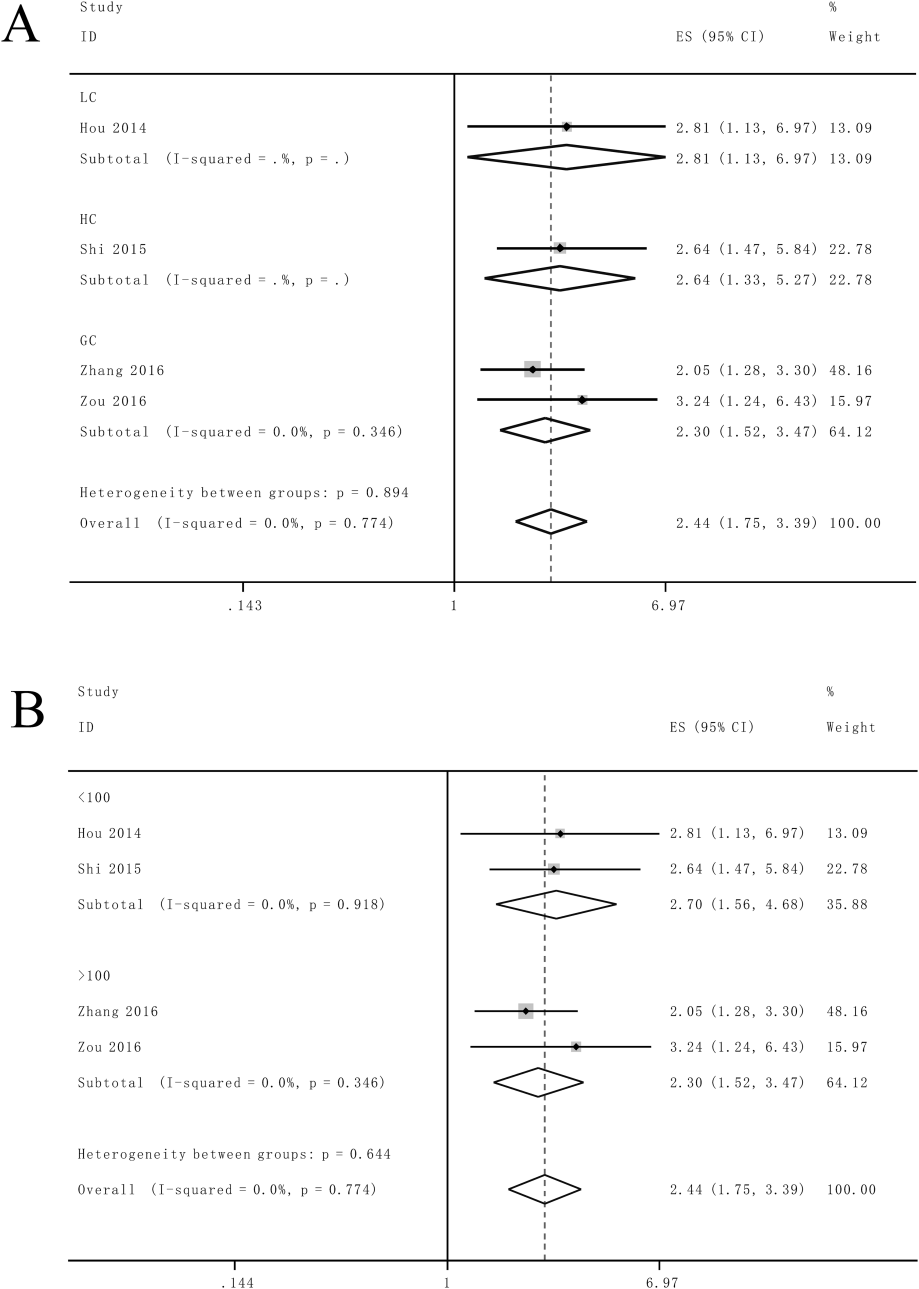
Stratified analyses for the association between SOX2OT expression with overall survival (OS). (A) Subgroup analysis of HRs of OS by factor of cancer type. (B) Subgroup analysis of HRs of OS by factor of sample size.

Afterwards the stratified analyses were performed by factor of cancer types and sample sizes to further analyze the data. For studies evaluating OS in different type of cancer, the results suggested that promoted SOX2OT levels could estimate worse outcome in gastric cancer (HR = 2.30, 95% CI: 1.52–3.47, *P*<0.0001) (Fig 4A). Furthermore, we found sample sizes did not alter the predictive value of SOX2OT on OS in various cancers (Fig 4B). No significant heterogeneity was detected in the subgroups analysis.

**Fig 5:**
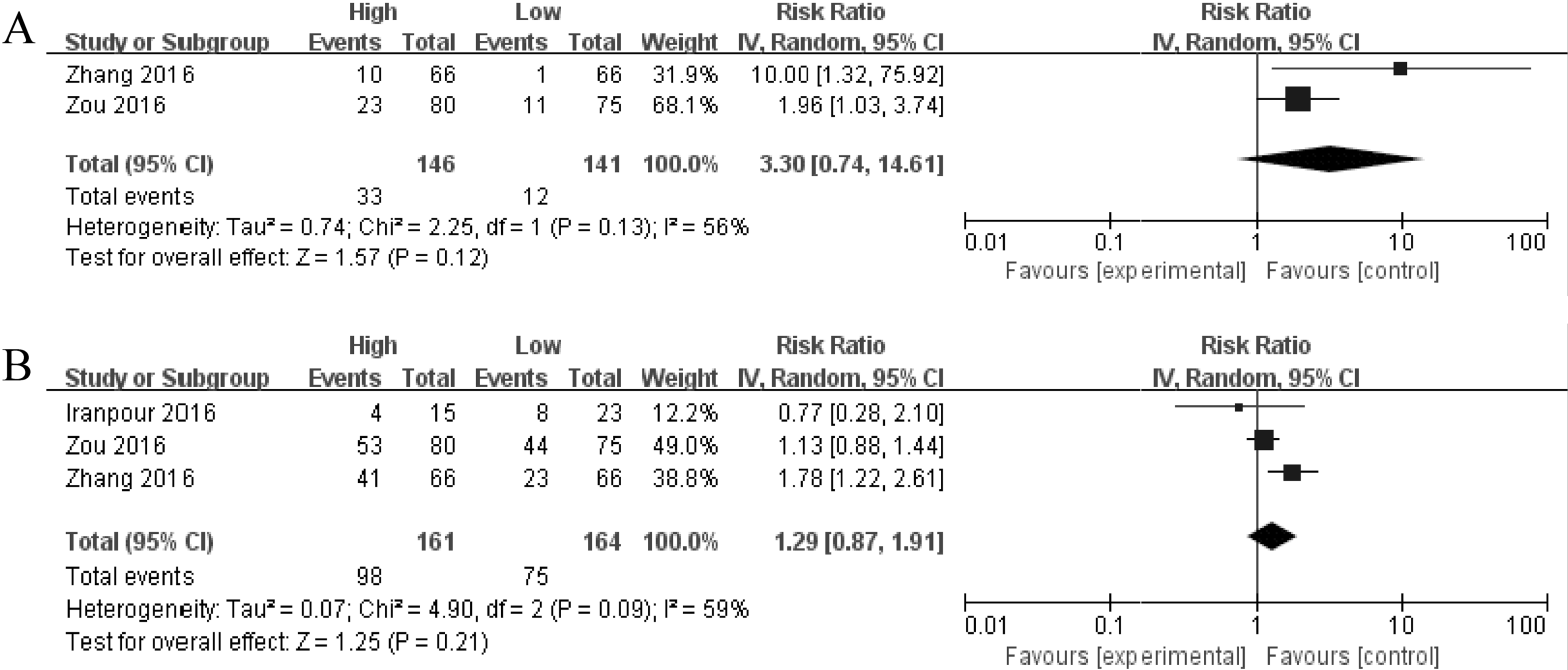
Forest plot for the association between SOX2OT expression with distant metastasis (A) and lymph node metastasis (B).

Subsequently, we set out to throw light upon the prognostic role of SOX2OT with distance metastasis and lymph node metastasis in various cancers. The characteristics of the involved studies which evaluating the association between SOX2OT levels with distance metastasis and lymph node metastasis were summarized in Fig 5. Through comparing the incidence of distance metastasis and lymph node metastasis between high and low SOX2OT expression group by random model, we found that patients with increased SOX2OT levels failed to show incline to distance metastasis (HR: 3.30, 95% CI: 0.74–14.61, *P* = 0.12) and lymph node metastasis (HR: 1.29, 95% CI: 0.87–1.91, *P* = 0.21), respectively.

### Publication bias

**Fig 6:**
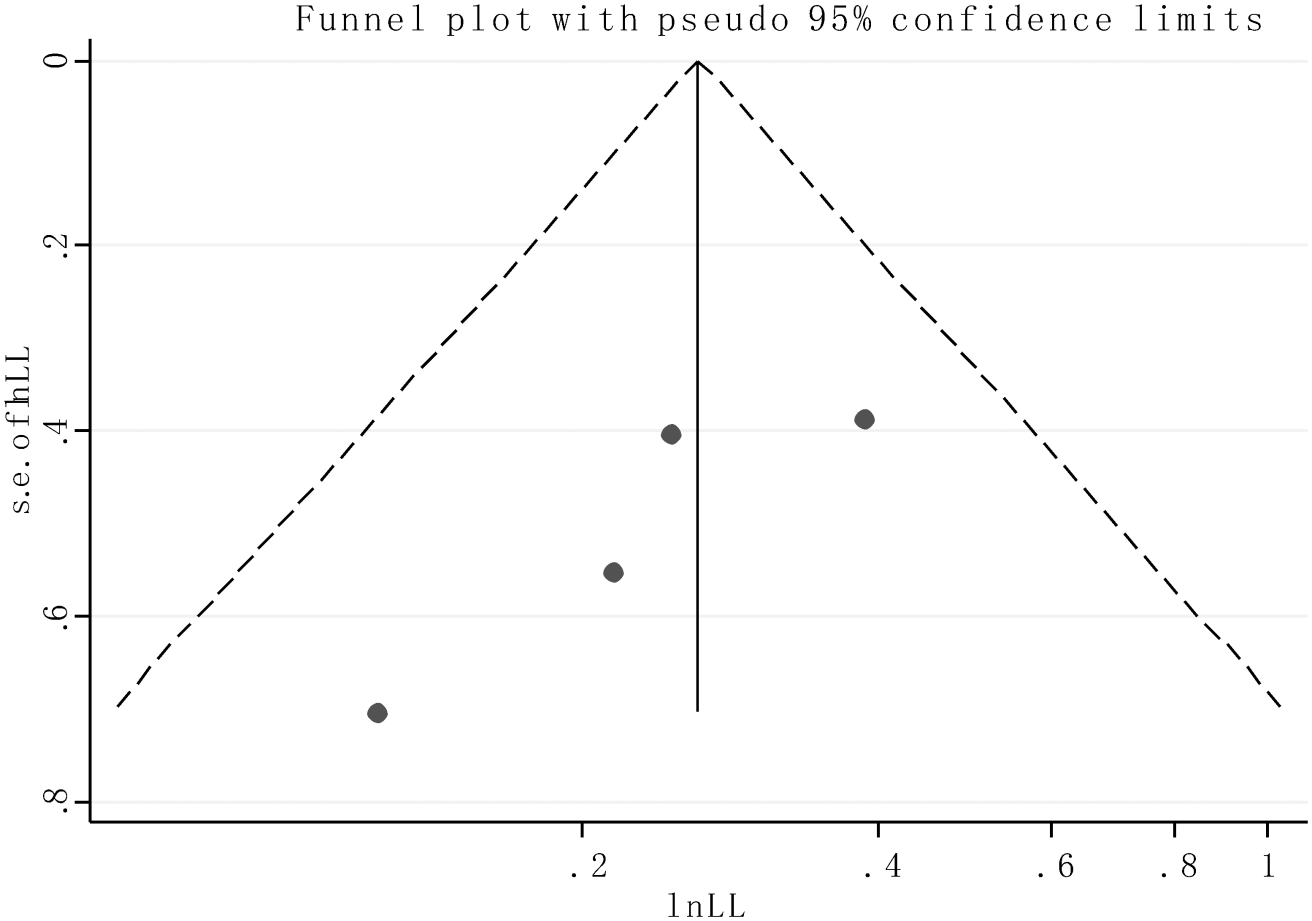
Funnel plot of the publication bias for overall survival.

Publication bias of the included articles was assessed by funnel plot and Begg’s bias test. As expected, the shape of the funnel plot was symmetrical and the *P* value of the Begg’s test was 0.308 for OS of all enrolled articles, suggesting the absence of significant publication bias in the meta analysis (Fig 6).

### Sensitivity analysis

**Fig 7:**
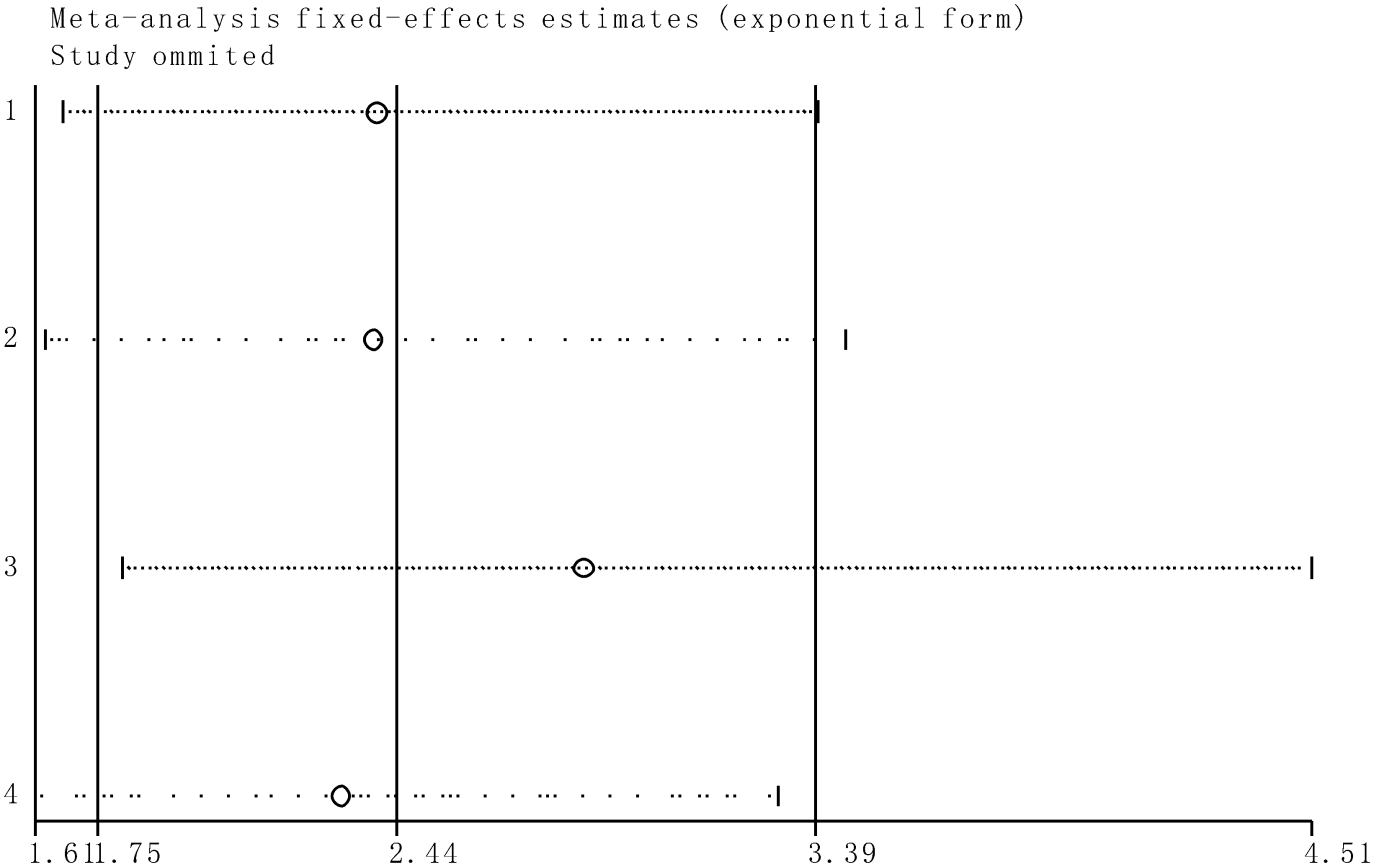
Sensitivity analyses of studies concerning SOX2OT and overall survival.

Sensitivity analysis indicated that the conclusions are stable because the pooled HRs was not significantly affected by the exclusion of any single study (Fig 7).

## Discussion

Recently, as the functions of tumor-associated lncRNAs have gradually been characterized, it was demonstrated that lncRNAs may participate in the tumorigenesis and disease progression[21, 22]. Moreover, accumulating evidences have suggested that the dysregulated of cancer-specific lncRNAs could serve as novel prognostic biomarkers to more precisely evaluate the prognosis of different tumors[23]. One of these hot lncRNAs is cancer-associated SOX2OT.

The mechanism underlying the relationship between elevated SOX2OT expression and poor prognosis in cancer patients need to be further elucidated. The mapping of SOX2OT gene proposed a possibility for the regulation of neighboring overlapped SOX2 gene[24]. Close concomitant expression patterns of SOX2OT and SOX2 in stem cells and some human cancers, have also highlighted the possibility that transcriptional regulation of SOX2 by SOX2OT[25, 26]. Furthermore, SOX2OT exerts regulatory function in cell cycle progression[26]. Recent studies manifested that SOX2OT contribute to the tumor progression of lung cancer, esophageal cell cancer and breast cancer through its regulation cell cycle progression[17, 25]. Knockdown of Sox2OT expression suppressed cancer cell growth by inducing G2/M arrest, prohibiting S phase entry and inhibiting cell proliferation which correlated with reduced protein levels of Cdc2 and Cyclin B1 in human lung cancer cell lines[17].

These studies consistently suggest that lncRNA SOX2OT may serve as a pivotal regulator in tumorigenesis and cancer progression. Since most individual clinical studies assessing the association of SOX2OT with OS have been limited by small sample sizes and controversial results, the current comprehensive meta analysis was performed to examine the clinical prognostic role of SOX2OT in a variety of carcinomas. A total of 5 studies including 421 patients were included in this study, and the results suggested that promoted SOX2OT expression was significantly correlated with poor prognosis and tumor progression in patients with various types of cancer. The analysis showed a pooled HR was 2.44 (95%CI: 1.75–3.39, *P*<0.0001), 1.62 (95%CI: 1.30–2.02, *P*<0.0001), 3.30 (95% CI: 0.74–14.61, *P* = 0.12) and 1.29 (95% CI: 0.87–1.91, *P* = 0.21) for OS, tumor progression (III/IV vs. I/II), DM and LNM, respectively.

To our knowledge, the current study is the first meta analysis in summarizing the values of SOX2OT in the prognosis of various cancers. Nonetheless the results of the current study should be interpreted with cautions because there are several limitations should be considered. First, the number of included studies in this meta-analysis was only five which might weaken the reliability of our results. Second, prominent heterogeneity was detected in the metastasis analysis and maybe contaminate our results. The heterogeneity was probably due to the differences in the clinical characteristics of patients (country, tumor stage etc.), the cut off value of SOX2OT, the types of cancer and so on. Finally, priority must be given to the cut off definition of SOX2OT expression among different investigations, which is the principal issue need to be resolved before its clinical application.

In conclusion, despite the inherent limitations described above, it is preliminarily concluded that elevated SOX2OT is significantly associated with OS in cancer patients, and may be considered as a potential and promising unfavorable prognostic factor in human cancers. Since the tumors are heterogeneous in terms of cell populations that could affect the measurements of mRNA levels in the tissues, the circulating levels of SOX2OT may be a better biomarker. In the future, well designed large sample studies with specific cut off value will be necessary to verify and strengthen the prognostic role of SOX2OT in cancer patients.

### Appendix: PubMed search terms

#1 Search (((Cancer[MeSH Terms]) OR Carcinoma[MeSH Terms]) OR Tumor) OR Neoplasm

#2 Search (((Cancer[Title/Abstract]) OR Carcinoma[Title/Abstract]) OR Tumor[Title/Abstract]) OR Neoplasm[Title/Abstract]

#3 Search (((((Cancer[MeSH Terms]) OR Carcinoma[MeSH Terms]) OR Tumor) OR Neoplasm)) OR ((((Cancer[Title/Abstract]) OR Carcinoma[Title/Abstract]) OR Tumor[Title/Abstract]) OR Neoplasm[Title/Abstract])

#4 Search (((prognosis[Title/Abstract]) OR prognostic[Title/Abstract]) OR survival[Title/Abstract]) OR metastasis[Title/Abstract]

#5 Search SOX2OT[Title/Abstract]

#6 #3 #4 and #5

## Supporting information

S1 Table MOOSE checklist. (DOC)

S2 Table PRISMA checklist. (DOC)

S3 Table Evaluations of the qualities of the included studies based on the Newcastle-Ottawa scale. (DOCX)
