# Supplementary material for: Prognostic value of lncRNA SOX2OT for Chinese cancer patients: A meta-analysis
Source: PLoS One. 2017 May 10;12(5):e0176889. doi: 10.1371/journal.pone.0176889 (PMC5425198; doi:10.1371/journal.pone.0176889)
Supplement: S1 Table — (DOC) [file pone.0176889.s001.doc]

**Table S1**. MOOSE checklist

| **Reporting of background should include** | |
| --- | --- |
| Problem definition | Background (Page 3) |
| Hypothesis statement | Background (Page 3) |
| Description of study outcome(s) | OS, DFS, LNM,DM |
| Type of exposure or intervention used | Various cancer (Page 3) |
| Type of study designs used | Meta-analysis (Page 3) |
| Study population | Global (Page 3) |
| **Reporting of search strategy should include** | |
| Qualifications of searchers (eg, librarians and investigators) | Investigator(Page 4) |
| Search strategy, including time period included in the synthesis and keywords | Search strategy and selection criteria (Page 3-4) |
| Effort to include all available studies, including contact with authors | We contact authors and searched reference lists and citations (Page 4) |
| Databases and registries searched | Methods (Page 3-4) |
| Search software used, name and version, including special features used (eg, explosion) | IE 10 |
| Use of hand searching (eg, reference lists of obtained articles) | Search strategy and selection criteria (Page 4) |
| List of citations located and those excluded, including justification | Flow diagram in Figure 1. (Page 5) |
| Method of addressing articles published in languages other than English | Search strategy and selection criteria (Page 4) |
| Method of handling abstracts and unpublished studies | Method (Page 4) |
| Description of any contact with authors | Method (Page 4) |
| **Reporting of methods should include** | |
| Description of relevance or appropriateness of studies assembled for assessing the hypothesis to be tested | Method (Page 4) |
| Rationale for the selection and coding of data (eg, sound clinical principles or convenience) | Methods (Page 4) |
| Documentation of how data were classified and coded (eg, multiple raters, blinding, and interrater reliability) | Methods (Page 4) |
| Assessment of confounding (eg, comparability of cases and controls in studies where appropriate) | Methods (Page 4) |
| Assessment of study quality, including blinding of quality assessors; stratification or regression on possible predictors of study results | Methods (Page 4) |
| Assessment of heterogeneity | Methods (Page 4) |
| Description of statistical methods (eg, complete description of fixed or random effects models, justification of whether the chosen models account for predictors of study results, dose-response models, or cumulative meta-analysis) in sufficient detail to be replicated | Methods (Page 4) |
| Provision of appropriate tables and graphics | Methods and Results (Page 4) |
| **Reporting of results should include** | |
| Graphic summarizing individual study estimates and overall estimate | Figure 2，3，4，5，6 and 7 |
| Table giving descriptive information for each study included | Table 1 |
| Results of sensitivity testing (eg, subgroup analysis) | Meta-analysis (Page 5-6) |
| Indication of statistical uncertainty of findings | Discussion (Page 7) |
| **Reporting of discussion should include** | |
| Quantitative assessment of bias (eg, publication bias) | Results (Page 6) |
| Justification for exclusion (eg, exclusion of non–English-language citations) | Discussion (Page 7) |
| Assessment of quality of included studies | Quality Assessment (Page 4) |
| **Reporting of conclusions should include** | |
| Consideration of alternative explanations for observed results | Discussion (Page 7) |
| Generalisation of the conclusions (ie, appropriate for the data presented and within the domain of the literature review) | Discussion (Page 7) |
| Guidelines for future research | Discussion (Page 8) |
| Disclosure of funding source | Grant Support (Page 9) |
