# Supplementary material for: Prognostic value of lncRNA SOX2OT for Chinese cancer patients: A meta-analysis
Source: PLoS One. 2017 May 10;12(5):e0176889. doi: 10.1371/journal.pone.0176889 (PMC5425198; doi:10.1371/journal.pone.0176889)
Supplement: S3 Table — (DOCX) [file pone.0176889.s003.docx]

Table S3 Evaluations of the qualities of the included studies based on the Newcastle-Ottawa Scale

|  | **Selection** |  |  |  |  | **Comparability** |  | **Outcome** |  |  |  | **total** |
| --- | --- | --- | --- | --- | --- | --- | --- | --- | --- | --- | --- | --- |
| Author | 1)Representativeness of the exposed cohort | 2) Selection of the non-exposed cohort | 3) Ascertainment of exposure | 4) Demonstration that outcome of interest was not present at start of study |  | 1) Comparability of cohorts on the basis of the design or analysis |  | 1) Assessment of outcome | 2) Was follow-up long enough for outcomes to occur | 3) Adequacy of follow up of cohorts |  |  |
| Hou 2014 | * | * | * | * |  | ** |  | * | * | * |  | 9 |
| Shi 2015 | * | * | * | * |  | * |  | * | * | * |  | 8 |
| Zhang 2016 | * | * | * | * |  | ** |  | * | * | * |  | 9 |
| Zou 2016 | * | * | * | * |  | * |  | * | * | * |  | 8 |
